# 2,3,7,8-Tetrachlorodibenzo-*p*-Dioxin Promotes BHV-1 Infection in Mammalian Cells by Interfering with Iron Homeostasis Regulation

**DOI:** 10.1371/journal.pone.0058845

**Published:** 2013-03-08

**Authors:** Filomena Fiorito, Carlo Irace, Antonio Di Pascale, Alfredo Colonna, Giuseppe Iovane, Ugo Pagnini, Rita Santamaria, Luisa De Martino

**Affiliations:** 1 Dipartimento di Medicina Veterinaria e Produzioni Animali, Università degli Studi di Napoli “Federico II”, Naples, Italy; 2 Dipartimento di Farmacia, Università degli Studi di Napoli “Federico II”, Naples, Italy; Queen Mary University of London, United Kingdom

## Abstract

Mammalian cells require iron to satisfy metabolic needs or to accomplish specialized functions, and DNA viruses, like bovine herpesvirus 1 (BHV-1), require an iron-replete host to efficiently replicate, so that iron bioavailability is an important component of viral virulence. Cellular iron metabolism is coordinately controlled by the Iron Regulatory Proteins (IRP1 and IRP2), whose activity is affected by 2,3,7,8-tetrachlorodibenzo-*p*-dioxin (TCDD), a current and persistent environmental contaminant. Considering that TCDD enhances BHV-1 replication, herein we analyzed the effects of TCDD on iron metabolism during BHV-1 infection in MDBK cells, and presented evidences of a divergent modulation of IRP1 and IRP2 RNA-binding capacity. Moreover, an up-regulation of transferrin receptor 1 (TfR1) and a concomitant down-regulation of ferritin were observed. This scenario led to an expansion of the labile iron pool (LIP) and induces a significant enhance of viral titer, as confirmed by increased levels of BHV-1 infected cell protein 0 (bICP0), the major transcriptional regulatory protein of BHV-1. Taken together, our data suggest that TCDD increases the free intracellular iron availability thereby promoting the onset of BHV-1 infection and rendering bovine cells more vulnerable to the virus.

## Introduction

Bovine herpesvirus 1 (BHV-1), an enveloped large double-stranded DNA virus of the Alphaherpesvirus family, is an important pathogen of cattle and the etiological agent of many types of disease, such as severe respiratory infection, conjunctivitis, genital disorders, abortions and shipping fever [1], [2]. Moreover, BHV-1 can establish latency in ganglionic neurons of the infected host and may predispose animals to secondary bacterial infections leading to pneumonia and occasionally to death [1]. Reactivation from latency can be stimulated by immunosuppression conditions, such as dexamethasone administration or increases in natural corticosteroids, resulting in virus spread to susceptible hosts [1]. Consequently, BHV-1 infections can provoke a significant economic loss in cattle industry.

Among a variety of biological targets, it is well established that the immune system is one of the most liable for 2,3,7,8-tetrachlorodibenzo-*p*-dioxin (TCDD), a persistent environmental contaminant that since 1997 has been classified as a cancer promoter [3]. TCDD causes a broad range of immunologic effects in experimental animals, including decreased host resistance to infectious disease and suppressed humoral and cell-mediated immune responses [4]. In particular, it has been demonstrated that TCDD exposure leads to an enhanced mortality in mice infected with influenza virus [5], increased gene expression of human immunodeficiency virus type-1 (HIV-1) in chronically infected cells [6], and an activation of cytomegalovirus replication in human fibroblasts [7]. Moreover, TCDD activates HIV-1 replication in a promyelocytic cell line latently infected with HIV-1 [8], and enhances BHV-1 replication in a bovine cell line [9].

More in detail, TCDD is one of the congeners of polychlorodibenzodioxin, a vast family of organochlorides that are produced through a variety of manufacturing and incineration processes. In the last years, levels of TCDD, exceeding the European Union tolerance, were detected in dairy products and milk from cow and water buffalo, raised on some areas of Campania Region (South Italy) [10]–[12], where BHV-1 is widespread and its eradication still represents a goal [13], [14].

The essential role of iron in the catalytic center of the DNA viruses ribonucleotide reductase give good reason for their need of an iron-replete host to efficiently replicate; as a result iron bioavailability become an important component of virulence [15], [16]. Indeed, some viruses selectively infect iron-acquiring cells by binding to transferrin receptor 1 during cell entry. Accordingly, changes in iron homeostasis seem to be involved in the pathogenesis of several virus infections and potential manipulations of host iron status may induce alterations in the virulence of many microorganisms [17]–[19].

Iron plays an essential role in cell life. Many proteins involved in vital physiological processes, including DNA synthesis, oxygen transport and cell respiration, need iron to function [20]. Cellular iron deficiency abrogates the activity of iron-dependent proteins and disrupts the proper functioning of cellular processes, causing cellular growth arrest and death [21]–[23]. Conversely, excess of iron is toxic, primarily by increasing oxidative stress and reactive oxygen species, which in turn cause the oxidation of lipids, proteins, and nucleic acids [24]–[26]. Since both iron deficiency and iron overload are detrimental to the cell, iron homeostasis must be tightly controlled by the coordinated and balanced expression of proteins involved in uptake, export, storage, and utilization [24], [27]. In fact, at cellular level, iron metabolism is coordinately controlled by the binding of Iron Regulatory Proteins (IRP1 and IRP2) to *cis*-regulatory mRNA motifs termed IREs; IRE/IRP interactions regulate the expression of the mRNAs encoding proteins for iron acquisition, such as transferrin receptor 1 (TfR1) and divalent metal transporter 1 (DMT1), and storage, such as ferritin [28], [29]. The labile iron pool primarily regulates RNA-binding capacity by IRP1 [4Fe-4S] cluster assembly/disassembly and by IRP2 degradation [30], [31]. However, the IRPs RNA-binding activity is also regulated by several other exogenous and endogenous factors, such as oxidative stress [32], nitric oxide signaling [33], protein phosphorylation [34], hypoxia/reoxygenation [35], viral infections [36], estrogens [37], TCDD exposure [38], as well as oxalomalic acid, an inhibitor of IRP1/c-aconitase [39].

Recently, we have provided the first evidence that TCDD impairs the maintenance of iron homeostasis in mammalian cells by modulating the expression and activity of the main cellular proteins involved in iron metabolism, leading to important changes in the cytosolic free iron (labile iron pool, LIP) [38]. Moreover, in a previous study we have shown that iron metabolism is critical for the interaction between BHV-1 and mammalian cells, where the association between host iron status and virus infectivity appears to be bidirectional: host iron status may alter the severity of disease as well as infection can alter cellular iron homeostasis [36]. Hence, considering that regulation of iron homeostasis is a critical point in the virus-host interaction, the current dioxin emergency [10]–[12], and the widespread diffusion of BHV-1 [13], [14], in this study we have investigated the iron metabolism during BHV-1 infection in the presence of TCDD in a bovine cell line, focusing on its possible impact on disease progression.

## Materials and Methods

### Cell cultures, virus infection and TCDD exposure

Madin – Darby Bovine Kidney (MDBK) cells (CCL22, American Type Culture Collection) were cultured in Dulbecco's modified Eagle's minimal essential medium (DMEM), supplemented with 2% foetal calf serum, 1% L-glutamine, 1% penicilline/streptomycine, 0.2% sodium pyruvate and 0.1% tylosine. All cultures were maintained in an incubator at 37°C (in 5% CO_2_/95% air). This cell line was maintained free of mycoplasma and of bovine viral diarrhoea virus.

The BHV-1 Cooper strain was used throughout the study. Virus stocks were routinely grown on MDBK cells and were also used for determination of virus titers [40].

2,3,7,8-Tetrachlorodibenzo-p-dioxin (TCDD) in toluene was purchased from Supelco (St. Louis, MO). The original TCDD concentration was 10 μg/ml, and it was initially diluted to give a 10,000 pg/ml stock solution by mixing with DMEM. This stock solution was then diluted to give working solutions of 0.01, 1 and 100 pg/ml in DMEM, which were added to cultures. All other chemicals were of the highest purity that is commercially available.

MDBK cells, at confluency, were washed with DMEM and then infected or not with BHV-1, at multiplicity of infection (MOI) of 1, in the presence or not of different concentrations of TCDD (0.01, 1 and 100 pg/ml). After 1 hr of adsorption at 37°C, the cells were incubated for indicated times post infection (p.i.) and then processed.

For iron repletion – depletion experiments, cells were treated with 50 μg/ml ferric ammonium citrate (FAC) or with 100 μM desferrioxamine (DFO) (Desferal, Novartis, Origgio, Varese, Italy) in growth medium for 18 h.

### Cell viability

Cell viability was evaluated by MTT assay. The principle of this method is that 3-(4,5-dimethyl-2-thiazolyl)-2,5-diphenyl-2H-tetrazolium bromide (MTT), a soluble tetrazolium salt, is converted to insoluble formazan by active mitochondrial dehydrogenases of living cells. Such conversion from yellowish soluble tetrazolium to purple formazan can be assayed spectrofluorimetrically. MDBK cells (2×10^4^ cells/well), in 96-well plates, at confluency, were infected or not with BHV-1, at MOI 1, in presence or in absence of TCDD (0.01, 1, or 100 pg/ml) and incubated for 12, 24 and 48 h. MTT (5 mg/ml) was added to cells and after further 4 h of incubation, the medium was removed and replaced with DMSO to solubilize the MTT formazan crystals. The spectrophotometer absorbance at 570 nm was determined. Data are calculated as a percentage of the control, and results are the mean ± SEM of four independent experiments performed in duplicate.

### Iron repletion-depletion and virus production

MDBK cells, at confluence, were infected with BHV-1 at MOI 1, in the presence or not of different concentrations of TCDD (0.01, 1 or 100 pg/ml). For iron repletion-depletion experiments, cells were treated respectively with FAC or DFO for 18 h, as above reported.

For the virus production assay, we analyzed both viral cytopathic effects (CPE) and virus titration. At 24 or 48 h p.i., all groups were observed under light microscope to evaluate CPE, represented by ample syncytia formation along with elimination of the cellular sheets.

For virus titration, at 24 or 48 h p.i., cell extracts, obtained by three cycles of freezing and thawing, were collected and stored in aliquots at −80°C. Virus titers were assayed by TCID_50_ method according to Reed and Muench [41].

To added further data about the virus production, we also evaluated protein levels of BHV-1 infected cell protein 0 (bICP0), the major transcriptional regulatory protein of BHV-1. After 48 h of infection, cells were collected by scraping and low-speed centrifugation and then processed with anti-bICP0 by Western Blot, as below described.

### Preparation of cellular extracts

After different times of BHV-1-infection (12, 24 and 48 h), in the presence or not of TCDD (0.01, 1 and 100 pg/ml), MDBK cells were washed and scraped off with PBS containing 1 mM EDTA. To obtain cytosolic extracts for electrophoretic mobility shift assay (EMSA), cells were treated with lysis buffer containing 10 mM HEPES, pH 7.5, 3 mM MgCl_2_, 40 mM KCl, 5% (v/v) glycerol, 1 mM dithiothreitol (DTT), 0.2% (v/v) Nonidet P-40 (NP-40) and protease inhibitor tablets (Roche, Mannheim, Germany) at 4°C. Cell debris and nuclei were pelleted by centrifugation at 15,000× *g* for 10 min at 4°C, and supernatants were stored at −80°C.

For Western blot analysis of IRPs, TfR-1, ferritin, DMT-1 and bICP0, cells were collected by scraping and low-speed centrifugation. Cell pellets were lysed at 4°C for 30 min in a buffer containing 20 mM Tris-HCl, pH 7.4, 150 mM NaCl, 5 mM EDTA, 5% (v/v) glycerol, 10 mM NP-40 and protease inhibitor tablets (Roche). The supernatant fraction was obtained by centrifugation at 15,000× *g* for 10 min at 4°C and then stored at −80°C. For Western blot analysis of the viral protein bICP0, cells were homogenized into lysis buffer (50 mM HEPES,150 mM NaCl, 1 mM EDTA, 1 mM EGTA, 10% glycerol, 1% Triton X-100, 1 mM phenylmethylsulfonyl fluoride, 1 mg/ml aprotinin, 0.5 mM sodium orthovanadate, and 20 mM sodium pyrophosphate). The lysates were clarified by centrifugation at 15,000× *g* for 10 min at 4°C and then stored at −80°C. Protein concentration was determined by the Bio-Rad protein assay (Bio-Rad, Milan, Italy).

### Electrophoretic mobility-shift assay (EMSA)

Plasmid pSPT-fer containing the sequence corresponding to the IRE of the H-chain of human ferritin, linearized at the BamHI site, was transcribed *in vitro* as previously described [42]. For RNA – protein band-shift analysis, cytosolic extracts (5 μg) were incubated for 30 min at room temperature with 0.2 ng of in vitro-transcribed ^32^P-labelled IRE RNA. The reaction was performed in buffer containing 10 mM HEPES, pH 7.5, 3 mM MgCl_2_, 40 mM KCl, 5% (v/v) glycerol, 1 mM DTT and 0.07% (v/v) NP-40, in a final volume of 20 μl. To recover total IRP1 binding activity, cytosolic extracts were pre-incubated for 10 min with 2-mercaptoethanol (2-ME) at 2% (v/v) final concentration, before the addition of ^32^P-labelled IRE RNA. Unbound RNA was digested for 10 min with 1 U RNase T_1_ (Roche), and non-specific RNA – protein interactions were displaced by the addition of 5 mg/ml heparin for 10 min. RNA – protein complexes were separated on 6% non-denaturing polyacrylamide gel for 2 h at 200 V. After electrophoresis, the gel was dried and autoradiographed at −80°C. The IRPs – IRE complexes were quantified with a GS-800 imaging densitometer (Bio-Rad). Concerning IRP1, the results are expressed as the percentage of RNA binding activity versus 2-mercaptoethanol treated samples; concerning IRP2 binding activity, results are expressed as percentage of untreated cells.

### Western blot analysis

Samples containing 50–100 μg of proteins were denatured, separated on a 12% (for ferritin and bICP0) or 8% (for IRP1, IRP2, TfR-1 and DMT-1) SDS-polyacrylamide gel and electro-transferred onto a nitrocellulose membrane (Amersham Biosciences, Little Chalfont, Buckinghamshire, UK) using a Bio-Rad Transblot (Bio-Rad). Proteins were visualized on the filters by reversible staining with Ponceau-S solution and destained in PBS. Membranes were blocked at room temperature in milk buffer [1× PBS, 5–10% (w/v) non-fat dry milk, 0.2% (v/v) Tween-20] and then incubated at 4°C overnight with 1∶1000 rabbit polyclonal antibody to human ferritin (Dako Cytomation, Glostrup, Denmark), or with 1∶1000 mouse monoclonal antibody to human transferrin receptor 1 (Zymed Laboratories Inc., CA), or with 1∶250 goat polyclonal antibody to human IRP1 (Santa Cruz Biotechnology, Santa Cruz, CA), or with 1∶250 goat polyclonal antibody to human IRP2 (Santa Cruz Biotechnology, Santa Cruz, CA), or with 1∶250 goat polyclonal antibody to human DMT-1 (Santa Cruz Biotechnology), or with 1∶800 polyclonal rabbit anti-bICP0 (a.a. 663–676) serum, kindly provided by Prof. M. Schwyzer (University of Zurich, Switzerland) [43]. Subsequently, the membranes were incubated for 90 min at room temperature with peroxidase-conjugated goat anti-rabbit IgG, or peroxidase-conjugated goat antimouse IgG+IgM, or peroxidase-conjugated rabbit anti-goat IgG (all the secondary antibodies were purchased from Jackson ImmunoResearch Laboratories, Baltimore Pike, West Grove, PA). The resulting complexes were visualized using chemoluminescence Western blotting detection reagents (ECL, Amersham Biosciences). The optical density of the bands was determined by a GS-800 imaging densitometer (Bio-Rad). Normalization of results was ensured by incubating the nitrocellulose membranes in parallel with the β-actin antibody.

### Cellular Labile Iron Pool (LIP) evaluation

The cellular labile iron content was estimated by a fluorimetric assay using the metalsensitive probe calcein (CA) [44] and the strong membrane-permeant iron chelator SIH (salicylaldehyde isonicotinoyl hydrazone), generously provided by Prof. Prem Ponka (McGill University, Montreal, QC, Canada). After incubation for 12, 24 and 48 h with 0.01, 1, or 100 pg/ml of TCDD, MDBK cells, plated at a density of 1.5×10^3^ cells/well, were loaded with 0.5 μM CAAM (calcein-acetomethoxy, Molecular Probes, Invitrogen, Eugene, OR) for 45 min at 37°C in calcium- and bicarbonate-free modified Krebs Henseleit buffer (KHB), consisting of 20 mM HEPES, pH 7.4, 119 mM NaCl, 4.9 mM KCl, 0.96 mM KH_2_PO_4_ and 5 mM glucose. CA-AM rapidly penetrates across the plasma membrane and is intracellularly hydrolysed to release free CA. After loading, the cultures were washed of excess CA-AM two times with KHB. Cellular CA fluorescence was recorded in a Perkin Elmer microplate reader (Perkin Elmer LS 55 Luminescence Spectrometer, Beaconsfield, UK) using a filter combination with an excitation wavelength of 485 nm and an emission wavelength of 530 nm (slits 5 nm). Cell cultures without CA-AM were used as blank to correct non-specific autofluorescence. Trypan blue was added in all experiments to eliminate extra-cellular fluorescence. Once hydrolyzed, calcein becomes trapped in the cytoplasm and emits intense green fluorescence. The calcein-loaded cells have a fluorescence component (ΔF) that is quenched by intracellular iron and can be revealed by addition of 100 μM SIH. The rise in fluorescence is equivalent to the change in calcein concentration or to the amount of cellular iron originally bound to CA. Thus, the changes in CA fluorescence intensity were directly proportional to the iron labile pool. To characterize the responsiveness of CA fluorescence toward different concentrations of intracellular iron, cells were preloaded with ferrous ammonium sulphate, ferric ammonium citrate or with the cell-permeable ferrous iron chelator SIH.

### Statistical analysis

For the MTT assay, cell counting and LIP determination, results are expressed as mean of percentage ± standard error of the mean (SEM) of n observations respect to control cells (100%), where n represents the number of experiments performed on different days. The results were analyzed by one-way ANOVA followed by a Bonferroni post hoc test for multiple comparisons. A *p*-value less than 0.05 was considered significant. The densitometric data from EMSA and Western blot analysis are reported as percentage of controls ± standard error of the mean (SEM) of n observations, where n represents the number of experiments performed on different days. Statistical significance among the results was determined by the ANOVA followed by the Newman-Keuls test. A *p*-value less than 0.05 was considered statistically significant.

## Results

### Effect of TCDD exposure during BHV-1 infection on MDBK cells viability

In order to evaluate the effect of TCDD on cell viability during BHV-1 infection, MDBK cells were infected with BHV-1, at a multiplicity of infection (MOI) of 1, alone or in association with different concentrations of TCDD (0.01, 1 or 100 pg/ml) and underwent, at different hours post infection, to MTT assay, as described in Method section.

**Figure 1:**
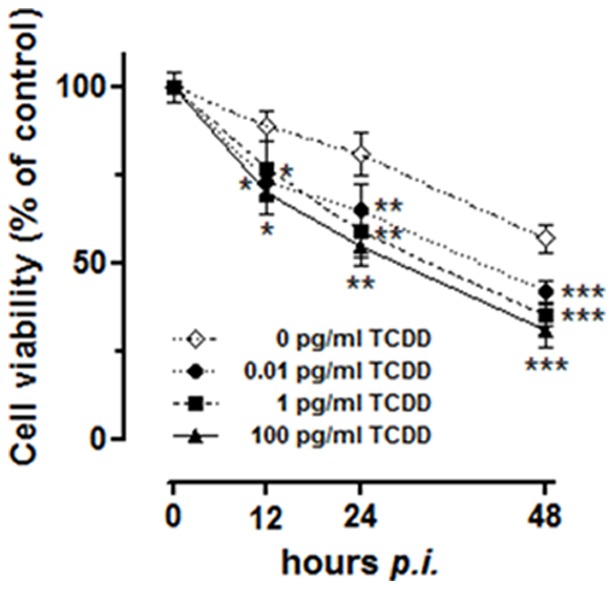
TCDD effect on MDBK cells viability during BHV-1 infection. Cell viability evaluated by an MTT assay procedure on MDBK cultures incubated or not with different TCDD concentrations (0.01, 1, 100 pg/ml) for various times post-BHV-1 infection (12, 24 and 48 hours *p.i.*) at a MOI of 1, as indicated in the legends. Results are expressed in graph as the percentage of untreated control cells and are reported as mean of six independent experiments ± SEM. * *p*<0.1 *vs* 0 pg/ml TCDD; ** *p*<0.01 *vs* 0 pg/ml TCDD; *** *p*<0.001 *vs* 0 pg/ml TCDD.

TCDD induced a significant decrease in viability of BHV-1 infected groups, as compared to unexposed controls (Fig. 1). In particular, after 12 h p.i., we observed a significant (*p*<0.05) reduction of cell viability in the presence of the all doses of TCDD tested. From 24 to 48 h p.i., TCDD drastically and significantly (*p*<0.01 and *p*<0.001) reduced cell viability of infected cells in a dose-dependent manner.

### Effect of TCDD exposure during BHV-1 infection on iron regulatory proteins activity and expression in MDBK cells

**Figure 2:**
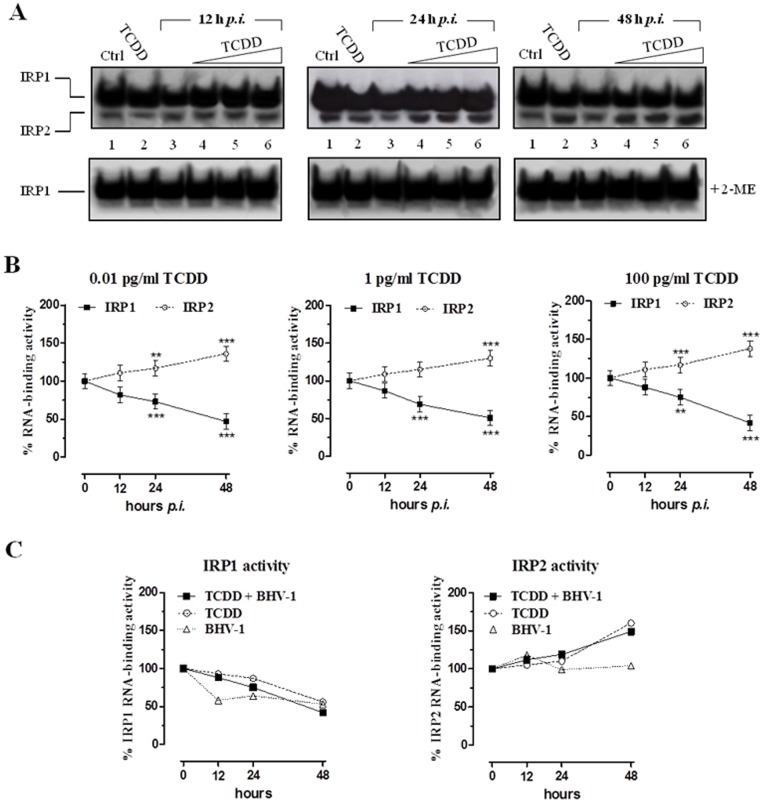
TCDD effect on Iron Regulatory Proteins activity. (**A**) MDBK cells were treated with different concentrations of TCDD (0.01, 1, 100 pg/ml) for different times post-BHV-1 infection (12, 24 and 48 hours *p.i.*) at a MOI of 1, as indicated in lanes 4, 5, and 6 of each gel. Controls cells were treated with 100 pg/ml TCDD alone or infected with BHV-1 at a MOI of 1 (lanes 2 and 3, respectively, of each gel) for the indicated times (12, 24, and 48 h). Proteins were extracted and subjected to electrophoretic mobility-shift assay (EMSA). RNA band-shift assay was performed with 5 μg of cytoplasmic proteins and an excess of ^32^P-labeled IRE probe in absence (top) or presence (bottom) of 2% 2-ME. RNA-protein complexes were separated on non-denaturing 6% polyacrylamide gels and revealed by autoradiography. The autoradiograms shown are representative of four experiments. (**B**) IRP1-RNA and IRP2-RNA complexes were quantified by densitometric analysis and results of EMSA experiments performed without 2-ME were plotted in graphs as percent of the control and are the average ± SEM values of four independent experiments (solid line IRP-1; dotted line IRP-2). Graphs are distinctive depending by the indicated TCDD concentrations. ** *p*<0.01 compared with controls; *** *p*<0.001 compared with controls. (**C**) Comparison between the effects of BHV-1 infection and TCDD exposure, taken individually or in combination, on IRPs activity plotted in graphs as percent of untreated control cells and by means of the 100 pg/ml dose of TCDD and the MOI of 1 for BHV-1 infection.

To investigate the effects of the concomitant TCDD exposure and BHV-1 infection on cellular iron metabolism in MDBK cells, we first analyzed the RNA-binding activity of IRPs. To this aim, confluent cultures of MDBK cells were infected with BHV-1 at multiplicity of infection (MOI) of 1 and exposed to different concentrations of TCDD (0.01, 1 and 100 pg/ml, lanes 4–6 of each experiment depicted in Fig. 2A, respectively) for 12, 24 and 48 h, and then the RNA binding activity was evaluated on cell lysates by means of EMSA. In order to better understand the combined effects of the viral infection and dioxin exposure on the regulation of iron metabolism, IRPs RNA-binding capacity was also evaluated in cells exposed for 12, 24 and 48 h to TCDD alone at the concentration of 100 pg/ml or infected for 12, 24 and 48 h with BHV-1 at a MOI of 1, as showed in lanes 2 and 3, respectively (Fig. 2A). In the light of our previous report showing that the dioxin effects on iron metabolism are independent of the dose [38], herein we decided to use TCDD at the single dose of 100 pg/ml as a control for the combined TCDD/BHV-1 treatment.

In the presence of both viral infection and dioxin, the results revealed a divergent regulation of IRPs activity, already evident at 12 h of treatment and at the lowest dose (0.01 pg/ml) of TCDD. In particular, as shown in Fig. 2A and 2B, the contemporary TCDD exposure and BHV-1 infection caused a significant time-dependent decrease in IRP1 RNA-binding activity with a reduction of about 55% with respect to control after 48 h at 100 pg/ml of TCDD treatment. A concomitant increase in IRP2 RNA-binding activity was also observed, which reached a maximum (∼145% vs. control) after 48 h of 100 pg/ml TCDD treatment. As clearly shown in the graphs in Fig. 2C by comparing between the effects of BHV-1 infection and TCDD exposure taken individually or in combination, the increase in IRP2 RNA-binding activity was exclusively induced by the dioxin, whereas the decreasing trend in IRP1 activity was induced by both BHV-1 and dioxin alone [36], [38].

**Figure 3:**
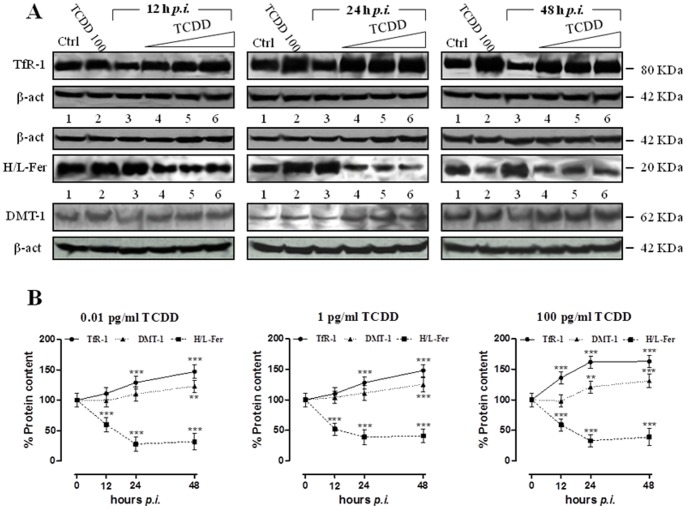
TCDD effect on iron regulatory proteins activity and expression in MDBK cells during BHV-1 infection. (**A**) Western blot analysis showing the IRP1 and IRP2 protein content in MDBK cells incubated with different TCDD concentrations (0.01, 1, 100 pg/ml) for different times post-BHV-1 infection (12, 24 and 48 hours *p.i.*), as indicated in lanes 4, 5, and 6 of each blot. Controls cells were treated with 100 pg/ml TCDD alone or infected with BHV-1 at a MOI of 1 (lanes 2 and 3, respectively, of each blot) for the indicated times (12, 24, and 48 h). Shown are blots representative of four independent experiments. Equal amounts of proteins (100 µg) were separated on a 8% SDS – polyacrylamide gel and subjected to Western blot analysis using 1∶250 dilution of IRP1 and IRP2 antisera. (**B**) After chemoluminescence, the corresponding bands were quantified by densitometric analysis and plotted in graphs as percentage of control in relation to the used TCDD concentrations, as indicated. The anti-β-actin antibody was used to standardize the amounts of proteins in each lane. Shown are the average ± SEM values of four independent experiments (solid line IRP-1; dotted line IRP-2).

Inclusion of 2% 2-mercaptoethanol (2-ME) in the EMSA experiments to convert all IRP1 to the RNA-binding form, showed any significant variation in total IRP1 RNA-binding activity suggesting that the observed inactivation of IRP1 was not due to a alterations in IRP1 protein content. These results were confirmed by the immunoblot analysis (Fig. 3), where no appreciable variations in the amounts of IRP1 and IRP2 proteins were observed, indicating that TCDD exposure and viral infection did not alter the IRP1 and IRP2 expression.

### Effect of TCDD exposure during BHV-1 infection on ferritin, TfR-1 and DMT1 expression in MDBK cells

**Figure 4:**
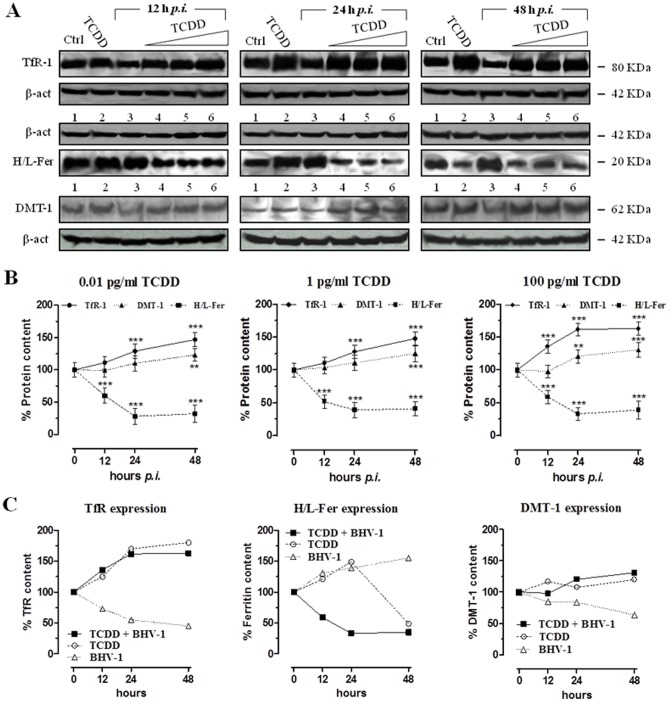
TCDD effect on ferritin, TfR-1 and DMT1 expression in MDBK cells during BHV-1 infection. (**A**) Western blot analysis showing the transferrin receptor-1 (TfR-1), the ferritin (H/L-Fer), and the divalent metal transporter-1 (DMT-1) levels in MDBK cells incubated with different TCDD concentrations (0.01, 1, 100 pg/ml) for different times post-BHV-1 infection (12, 24 and 48 hours *p.i.*), as indicated in lanes 4, 5, and 6 of each blot. Controls cells were treated with 100 pg/ml TCDD alone or infected with BHV-1 at a MOI of 1 (lanes 2 and 3, respectively, of each blot) for the indicated times (12, 24, and 48 h). Shown are blots representative of four independent experiments. For TfR-1 content analysis, equal amounts of cytosolic lysates containing 100 μg of proteins were fractionated by 8% SDS-PAGE and subjected to Western blot analysis using 1∶1000 dilution of TfR-1 antiserum. For ferritin content analysis, equal amounts of cytosolic lysates containing 100 μg of proteins were fractionated by 12% SDS-PAGE and subjected to Western blot analysis using 1∶1000 dilution of ferritin antiserum. For DMT-1 level analysis, equal amounts of cytosolic lysates containing 100 μg of proteins were fractionated by 8% SDS-PAGE and subjected to Western blot analysis using 1∶250 dilution of DMT-1 antiserum. The anti-β-actin antibody was used to standardize the amounts of proteins in each lane. (**B**) TfR-1, H/L-Fer and DMT-1 bands detected by chemoluminescence were quantified by densitometric analysis and plotted in graphs as percentage of control (solid line TfR-1; broken line ferritin; dotted line DMT-1) in relation to the used TCDD concentrations, as indicated. Shown are the average ± SEM values of four independent experiments. ** *p*<0.01 *vs* control cells; *** *p*<0.001 *vs* control cells. (**C**) Comparison between the effects of BHV-1 infection and TCDD exposure, taken alone or in combination, on the transferrin receptor-1 (TfR-1), the ferritin (H/L-Fer), and the divalent metal transporter-1 (DMT-1) levels in MDBK cells plotted in graphs as percent of untreated cells and by means of the 100 pg/ml dose of TCDD and the MOI of 1 for BHV-1 infection.

To evaluate the expression of proteins regulated at post-transcriptional level by IRPs, we examined levels of ferritin, TfR-1 and DMT1 by immunoblot analysis on lysates obtained from MDBK cells treated for 12, 24 and 48 h with 100 pg/ml TCDD or infected with BHV-1 at a MOI of 1 for 12, 24 and 48 h (Fig. 4A, lanes 2 and 3, respectively), and from cells simultaneously infected by BHV-1 (MOI of 1) and exposed for 12, 24 and 48 h to TCDD (0.01, 1 and 100 pg/ml). As depicted in Fig. 4A and in the corresponding graphs (4B), viral infection and TCDD exposure jointly induced a progressive increase of TfR-1 levels and a fast reduction of ferritin content. In fact, it was observed a statistically significant 1.5-fold increase of TfR-1 expression respect to control cells after 48 h of viral and TCDD exposure (0.01 and 1 pg/ml), whereas in the case of TCDD exposure at a dose of 100 pg/ml a further raise was evidenced already after 24 hours. Regarding ferritin, although a decrease is clearly detectable already after 12 h, the protein expression was dramatically decreased of about 60% with respect to control cells starting from 24 h of viral infection and TCDD exposure (at all used TCDD concentrations). TfR-1 and ferritin levels detected in MDBK cells in the presence of both viral infection and dioxin are similar to those observed in the presence of dioxin alone, especially concerning the trend of cellular TfR-1 content during treatments (see Fig. 4C). However, the overall reduction in ferritin protein levels caused by BHV-1 infection and TCDD in combination reproduced the decrease in ferritin content observed at the endpoint of the biphasic response originated by TCDD alone [38]. As concerns the expression of DMT1, immunoblot analysis has evidenced a trend of increase with a statistically significant 1.3-fold increase respect to control cells at 48 h at all tested dioxin doses. This trend also reflected that observed in the presence of dioxin alone.

### Cellular Labile Iron pool in MDBK cells after BHV-1 infection and TCDD exposure

**Figure 5:**
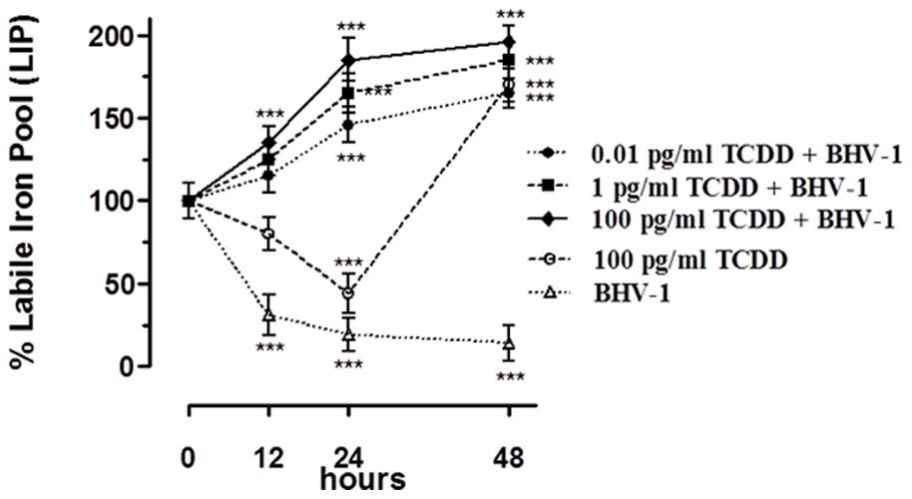
TCDD effect on Cellular Labile Iron pool in MDBK cells after BHV-1 infection. LIP extent variations in MDBK cells incubated with different TCDD concentrations (0.01, 1, 100 pg/ml) for 12, 24 and 48 hours, and/or infected with BHV-1 at a MOI of 1, estimated with the CA fluorescent method. Cell cultures were loaded with CA using acetomethoxyl-calcein, and fluorescence was measured before and after the addition of 100 mM permeant iron chelator SIH. Shown are the average ± SEM (n = 6) values of three independent experiments plotted in a graph as percent of control untreated cultures. *** *p*<0.001 *vs* control cells.

To verify whether the concurrent effects of BHV-1 infection and TCDD exposure on the expression of the main proteins implicated in iron metabolism could ultimately lead to variations in the free cellular iron content, we measured LIP amount using the calcein fluorimetric assay. The results obtained in BHV-1 infected MBDK cells, treated for 12, 24 and 48 h with 0.01, 1 and 100 pg/ml of TCDD, are represented in Fig. 5 and demonstrate that, in contrast to what occurred in the presence of the viral infection alone, the LIP progressively increased at all used TCDD concentrations, reaching the maximum after 48 h (about the 180% respect to untreated cells) when 100 pg/ml of TCDD was used. In agreement with the trend of ferritin protein content, the exposure to TCDD alone resulted in an early LIP decrease followed by a fast increase that reached the maximum after 48 h (about the 160% respect to untreated cells). Overall, the effects of the simultaneous TCDD exposure and BHV-1 infection on LIP extent correlate with the up-regulation of TfR-1 and the down-regulation of ferritin observed in MBDK cells during BHV-1-infection and TCDD exposure.

### Effect of iron repletion-depletion on virus production

To verify whether the expansion of LIP, caused by the concomitant BHV-1 infection and TCDD exposure, could play a role in virus production, we next analyzed both viral cytopathic effects and virus titration under different conditions of iron availability. To this aim, iron repleted or depleted MDBK cell cultures (by means of FAC or of DFO, respectively) were infected with BHV-1 and exposed or not to all the tested doses of TCDD and at last processed as reported in Method Section.

**Figure 6:**
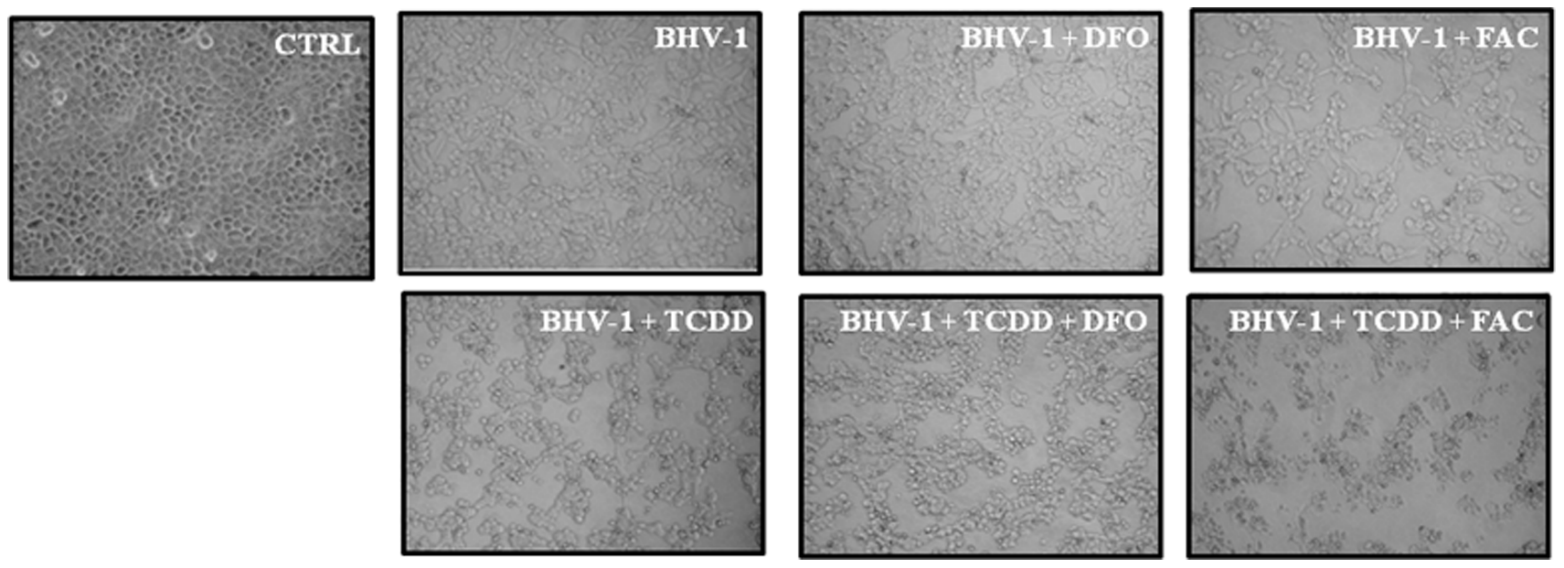
Effect of iron repletion-depletion on CPE. Representative microphotographs by phase-contrast light microscopy of iron deplete-replete MDBK cells exposed or not, as indicated in the legends, to 1 pg/ml of TCDD at 48 h post-BHV-1 infection (MOI of 1), showing the cytopathic effects and the morphological changes on cellular monolayers. In iron depletion – repletion experiments, cells were treated with 100 μM desferrioxamine (DFO) or with 50 μg/ml ferric ammonium citrate (FAC).

**Figure 7:**
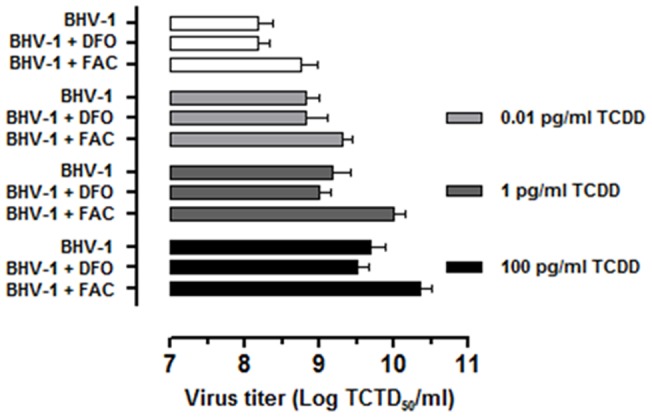
Effect of iron repletion-depletion on virus titer. Virus titers, assayed by TCID_50_ method and reported as Log TCID_50_/ml, in iron deplete-replete MDBK cells exposed to 0.01, 1, 100 pg/ml of TCDD at 48 h post-BHV-1 infection (MOI of 1), as indicated in the legends. In iron depletion – repletion experiments, MDBK cells were treated with 100 μM desferrioxamine (DFO) or with 50 μg/ml ferric ammonium citrate (FAC).

The CPE, represented by ample syncytia formation along with elimination of the cellular sheet, was always much evident in FAC treated cells after 48 h p.i. (Fig. 6). Virus titers, assayed by TCID50 method, confirmed the above data. In fact we evidenced a statistically significant increase of viral production in all FAC treated groups after 48 h p.i., and particularly in TCDD exposed cells (Fig. 7).

**Figure 8:**
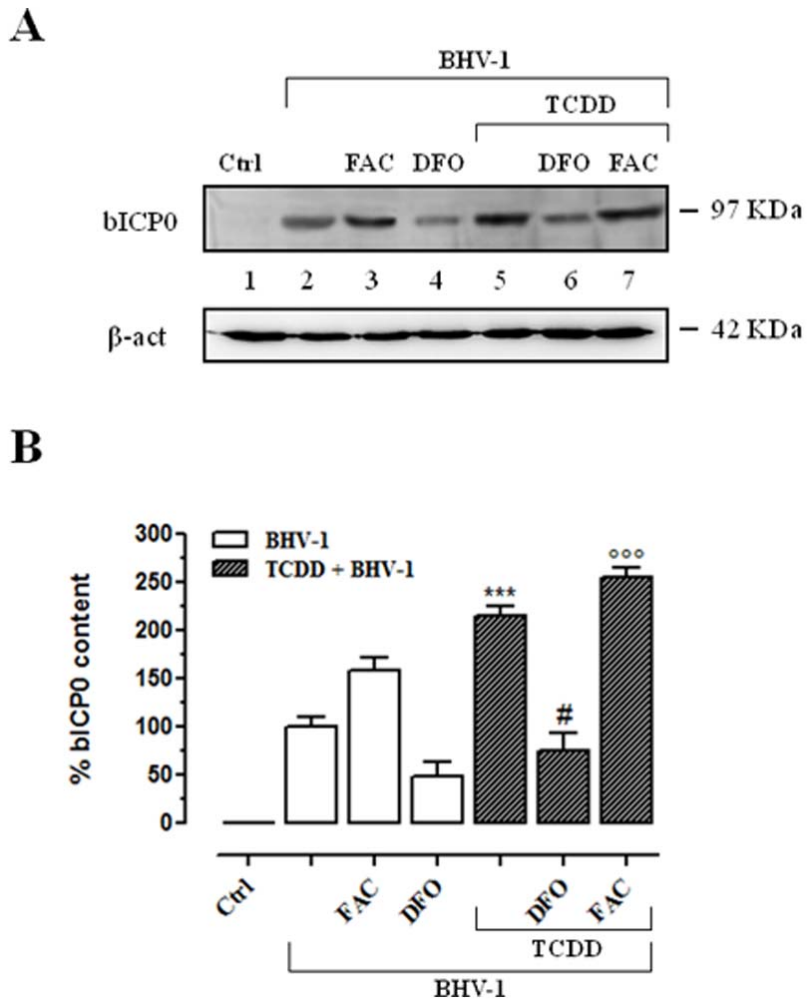
Effect of iron repletion-depletion on bICP0 protein levels. (**A**) Western blot analysis showing the bICP0 content in iron deplete-replete MDBK cells at 48 h post-BHV-1 infection (MOI of 1), in presence or not of 1 pg/ml of TCDD, as indicated in the legend. In iron depletion – repletion experiments, cells were treated with 100 μM desferrioxamine (DFO) or with 50 μg/ml ferric ammonium citrate (FAC). Equal amounts of cytosolic lysates containing 100 μg of proteins were fractionated by 12% SDS-PAGE and subjected to Western blot analysis using 1∶800 dilution of bICP0 polyclonal rabbit antiserum. The anti-β-actin antibody was used to standardize the amounts of proteins in each lane. Shown is a blot representative of four independent experiments. (**B**) bICP0 protein bands detected by chemoluminescence were quantified by densitometric analysis and plotted in bar graphs as percentage of control (open bars for iron deplete-replete BHV-1 infected cells, filled bars for iron deplete-replete BHV-1 infected cells exposed to TCDD, as indicated in the legend). Shown are the average ± SEM values of four independent experiments. *** *p*<0.001 *vs* BHV-1 infected cells; # *p*<0.1 *vs* iron depleted BHV-1 infected cells; °°° *p*<0.001 *vs* iron repleted BHV-1 infected cells.

To added further data about the virus production, in the same experimental conditions we also evaluated the levels of BHV-1 infected cell protein 0 (bICP0), the major transcriptional regulatory protein of BHV-1. As depicted in the Fig. 8 (lanes 5–7), TCDD induced an increased expression of bICP0 protein, in agreement to our previous studies on the effects of TCDD on bICP0 expression [45]. When cells were iron repleted (Fig. 8, lanes 3 and 7) a further increase in bICP0 protein levels was observed in BHV-1 infected cells but especially in infected/TCDD exposed cells. On the contrary, when cells were iron depleted a marked decrease in bICP0 protein content was always observed (Fig. 8, lanes 4 and 6).

## Discussion

Many proteins that exert fundamental cellular operations, including DNA synthesis, oxygen transport and cell respiration, need iron to function. Iron is widely used in nature as it serves as a prosthetic group for a number of enzymatic functions requiring electron transfer via oxidation – reduction reactions [20]. As a result, virtually all cells require iron for survival as highlighted by an imposing list of iron-containing enzymes that encompasses proteins of vital physiological significance [46]. Both at the systemic and the cellular level, the expression and the activity of proteins involved in iron metabolism are subjected to a fine regulation by many pathophysiological conditions. In addition, a range of exogenous factors can influence the iron homeostasis. Among these, dioxins, and in particular the environmental contaminant 2,3,7,8-tetrachlorodibenzo-*p*-dioxin (TCDD), can play a detrimental function on iron metabolism [47].

Indeed, we have recently provided the first evidence that TCDD impairs iron homeostasis in mammalian cells by modulating IRPs RNA-binding activity and other iron-related proteins, leading ultimately to an enlarged cellular iron pool [38]. Because of their hazard and wide distribution, dioxins and by-products are environmental toxins of current interest. In particular, TCDD is classified as a cancer promoter [3] and various molecular mechanisms have been suggested to be active in TCDD tumor induction [48]. Besides being carcinogenic, dioxins induce a broad spectrum of toxic and biological responses including immune suppression and increased susceptibility to infectious agents. With reference to microbial infections, we have already shown that the regulation of iron metabolism is critical for the interaction between DNA viruses, such as BHV-1, and mammalian cells [36], as well as Fiorito et al. have demonstrated that TCDD exposure enhances BHV-1 replication in bovine cells [45]. Considering the concomitant widespread environmental diffusion of both dioxin and BHV-1, within a broader research project aimed to investigate the biological effects of dioxin in mammalian cells, herein we have explored the combined effects of TCDD exposure and BHV-1 infection on cellular iron metabolism. In particular, we focused on the possible impact of TCDD-dependent iron metabolism dysregulation on the progression of virus infection in MDBK cells, a useful and standardized epithelial-like *in vitro* model to study both virus replication and TCDD exposure in mammalian cells [9], [38], [45], [49]–[51]. As well to get an insight into the regulation of cellular iron homeostasis in presence of environmental toxins, these studies may have important repercussions especially in those geographic areas where dioxin and BHV-1 are concomitantly widespread.

In agreement with our previous reports [9], [50], [51], in the present study we first confirmed that TCDD induce a significant time and dose dependent viability decrease of BHV-1 infected cells. This effect is likely linked to a cell increased susceptibility to viral infection induced by the dioxin exposure, as described later.

Concerning the effects on the iron metabolism caused by the concurrent BHV-1 infection and TCDD exposure, we observed a remarkable reduction of the IRP1 RNA-binding activity (by nearly 60% with respect to control cells after 48 h of treatment at 100 pg/ml). In contrast, the RNA-binding activity of IRP2 showed a significant and progressive increase compared to that of control cells; this increase became even more consistent when compared to the activity of IRP2 observed in the same cells treated with BHV-1 alone [36]. Overall, as it emerges by the comparison between the effects of BHV-1 infection and TCDD exposure taken separately or in combination, the divergent regulation of IRPs activity seems to be mainly a dioxin-dependent phenomenon. Indeed, in accordance to our previous findings, the increased activity of IRP2 was exclusively induced by TCDD exposure, whereas the decreased IRP1 activity was derived from both BHV-1 and TCDD [36], [38]. Therefore, TCDD seems to play a crucial role in the control of the RNA-binding capacity of IRP2 [38], also when a viral infection occurs, thus making IRP2 the major effector of cellular iron homeostasis when IRP1 fails in controlling iron sense. Interestingly, no appreciable variations in the amounts of IRP1 and IRP2 proteins were observed, indicating that TCDD exposure and viral infection, alone or in combination, interfere with their activity without affecting protein expression.

Because of IRPs central role within the control of iron metabolism, modulation of their binding activity leads to changes in iron related protein expression profiling. In fact, the cellular levels of TfR-1 and ferritin are influenced by the co-presence of dioxin and virus infection, resulting in a marked TfR-1 expression increase (of about 1.7-fold after 24 and 48 h of treatment at 100 pg/ml) at the plasma membrane and a concomitant reduction in ferritin content (by approximately 0.4-fold after 24 and 48 h of treatment at 100 pg/ml) compared to control cultures. The reduced ferritin levels coupled to the increased TfR-1 levels observed after TCDD and BHV-1 exposure are consistent with the increased IRP2 binding activity; in fact, IRP2 binds and stabilizes mRNA for TfR-1, thereby enhancing receptor synthesis. Interestingly, as previously stated with regard to the IRPs activity, the regulation of TfR-1 and ferritin expression in MDBK cells in the co-presence of viral infection and dioxin largely reflects that observed in the presence of dioxin alone, especially with reference to the cellular TfR-1 content. However, the reduction in ferritin protein levels caused by BHV-1 infection and TCDD in combination is consistent with the decrease in ferritin content observed at the endpoint of the biphasic response originated by TCDD alone [38], once again suggestive of the critical role of the dioxin in interfering with the maintenance of cellular iron homeostasis.

The modulation of the expression of the most important proteins involved in the maintenance of cellular iron homeostasis ultimately leads to a modification of LIP extent. In fact, the simultaneous exposure to TCDD and BHV-1 resulted in a important increase of LIP extension, causing an iron overload which could promote the onset of viral infection. In the same experimental conditions, the increased expression of DMT-1 is an additional contribution to increase the iron cellular up-take. The rapid and marked change in LIP levels strongly confirms the hypothesis that dioxin causes important alterations in the cellular iron homeostasis. This is a crucial point for the virus-host interplay. DNA viruses, such as herpes simplex virus 1, bovine herpesvirus 1 and vaccinia virus, like their human counterpart, are strictly dependent on iron for their proliferation as a result of the essential role that iron plays in the catalytic centre of ribonucleotide reductase [52]. Because BHV-1, such as other DNA viruses, have not evolved mechanisms for actively scavenging host iron [53], a competition for cellular iron between virus and host ribonucleotide reductase takes place throughout viral replication. When iron is accessible into the host cell, the viral ribonucleotide reductase permits virus replication by the sequestration of all available iron [54], [55]. Consequently, the enlarged intracellular iron pool promoted by dioxin exposure can make bovine cells more susceptible and vulnerable to virus infection.

On the basis of the overall results obtained by investigating the effects of BHV-1 infection and TCDD exposure, alone or in combination, we can state that the main factor that interferes with the regulation of cellular iron metabolism is represented by dioxin. Considering that iron status may alter the severity of viral infection disease, exposure to dioxin, which causes an enlarged free cytosolic iron pool, as well as triggering oxidative stress, seems to make cells more exposed to DNA viruses infection. Indeed, in iron-repleted cell cultures a marked increase in the levels of bICP0 – the major transcriptional regulatory protein of BHV-1 – was detected following BHV-1 infection. This effect was further enhanced by the presence of dioxin. On the contrary, when cells were iron-depleted, bICP0 protein content considerably decreased in the same experimental conditions. In accordance with previous studies carried out on viral infection and iron metabolism interaction [56], these results suggest that reducing iron availability to DNA virus should benefit the host.

In conclusion, altogether our observations highlight a central connection among iron homeostasis and viral infection during exposure to dioxins; the fine regulation of iron metabolism and the control of dynamic cellular processes during BHV-1 infection are further modified by the presence of TCDD. In particular, TCDD may act as an additional risk factor for disease progression *via* enhanced cellular free iron availability that render mammalian cells more susceptible to DNA virus infection.
